# Cervical spine kinematics after anterior cervical discectomy with or without implantation of a mobile cervical disc prosthesis; an RCT

**DOI:** 10.1186/s12891-015-0479-4

**Published:** 2015-02-21

**Authors:** Toon FM Boselie, Henk van Mameren, Rob A de Bie, Henk van Santbrink

**Affiliations:** Department of Neurosurgery, Maastricht University Medical Center, P. Debyelaan 25, 5800, 6202 AZ Maastricht, Netherlands; Department of Epidemiology, CAPHRI School for Public Health and Primary Care, Maastricht University, Maastricht, Netherlands

**Keywords:** Cervical disc arthroplasty, Cervical degenerative disc disease, Fusion, Cervical kinematics, Randomized controlled trial

## Abstract

**Background:**

When surgically treating cervical degenerative disc disease, the most commonly performed procedure is anterior cervical discectomy. This procedure is performed with, or without fusion promoting methods. For both options the rate of fusion is high and there is much debate whether fusion of the treated segment is a contributing factor to accelerated degeneration of adjacent motion segments. In an effort to prevent degeneration of adjacent segments (ASDeg) due to loss of mobility at the operated level, cervical disc arthroplasty (CDA) was introduced. To evaluate the effectiveness of CDA in preventing ASDeg long term studies are necessary. However, prevention of ASDeg is based on the premise that mobile disc prostheses preserve cervical spine motion in a physiological way. In this article the authors describe a short term protocol for a study that aims to investigate whether CDA reaches the intended goal: restoration or preservation of physiological cervical spine motion. To this end, a technique is used to establish the sequence of contributions of cervical motion segments to flexion/extension of the spine.

**Methods:**

24 subjects between 18 and 55 years old, with radicular symptoms due to a herniated disc between C5 and C7, refractory to conservative therapy are randomized to simple discectomy, or CDA. These groups are preceded by a pilot group of three subjects receiving CDA. Fluoroscopic flexion-extension recordings are acquired preoperatively, and at three and 12 months postoperative. At these same time points, patient reported outcomes are collected, and a neurological examination is performed by and independent physician.

**Discussion:**

Studies investigating arthroplasty determine mobility by measuring segmental range of motion (sROM), which gives no information other than presence, and quantity, of mobility. SROM suffer from high variability. The authors therefore chose to use a method previously used in healthy controls, to describe the dynamic process of cervical spine motion in more detail. Determining cervical spine motion patterns has been reported to be more consistent than sROM. If a physiological motion pattern is absent after surgery in the CDA group, prevention of future ASDeg is less likely. Radiological outcomes will be correlated to clinical outcomes.

**Trial Registration:**

NCT00868335

**Electronic supplementary material:**

The online version of this article (doi:10.1186/s12891-015-0479-4) contains supplementary material, which is available to authorized users.

## Background

Cervical degenerative disc disease is defined as complaints due to degenerative changes of the intervertebral disc and the intervertebral disc space. Herniation of the intervertebral disc itself or surrounding osteophytes cause radiculopathy due to compression of a nerve root, leading to pain and/or sensory and/or motor deficit. It can also lead to compression of the spinal cord itself causing myelopathy. Surgery can be considered if there is insufficient relief of symptoms with conservative treatment options, such as physical therapy, non-steroidal anti-inflammatory drugs, or immobilisation. Goal of surgery is relieving radiating arm pain in case of radiculopathy and prevention of progressive neurological deficits in case of myelopathy. The most commonly performed procedure is the anterior cervical discectomy, which is being performed either with, or without fusion promoting methods (called ‘fusion’ and ‘simple discectomy’, respectively). Clinical results of these methods are similarly good. Both methods have a high rate of fusion, up to 80% in simple discectomy, and over 95% in fusion [1-4].

There is much debate whether fusion is a contributing factor to accelerated degeneration of adjacent motion segments (adjacent segment degeneration, ASDeg) or not. When this degeneration causes complaints, it is called adjacent segment disease (ASDis). The annual risk of ASDis is reported to be 2.9% [5]. It is not clear if fusion is in fact the culprit, or if it is just progression of degeneration in an ageing spine [6-9].

In an effort to prevent degeneration of adjacent segments due to loss of mobility at the operated level, cervical disc arthroplasty (CDA) was introduced. In CDA, a mobile disc prosthesis is inserted during surgery to preserve mobility of the operated spinal segment. Short term clinical results for CDA are comparable to fusion, which’s results are comparable to simple discectomy [4,10,11]. Long term studies are necessary to evaluate their effectiveness in preventing ASDis [10,12].

Prevention of ASDis is based on the premise that mobile disc prostheses preserve cervical spine motion in a physiological way. Motion of the cervical spine is commonly measured by means of flexion-extension radiographs, which give information about the segmental range of motion (sROM). It suffers from a high intra- and inter-individual variability, severely limiting its use as a tool for diagnosis and follow-up in the individual patient. The sequence in which motion segments contribute to the movement to maximum extension or flexion in the entire cervical spine has been reported to be a more consistent parameter in healthy controls [13-15]. Therefore, a randomized controlled study to investigate the influence of implanting a mobile disc prosthesis on motion in the cervical spine was designed, using analysis of the sequence of segmental contributions.

Primary objective of the study is to ascertain whether a mobile cervical disc prosthesis restores/preserves the sequence of segmental contributions that is present in healthy controls, in contrast to patients undergoing simple discectomy. Secondary goal is to evaluate the clinical results of CDA with the prosthesis used, compared to simple discectomy, to determine if results are comparable to reported results for this type of surgery.

## Methods

### Subjects

Patients referred to our neurosurgical department who are eligible for surgery will be informed about the study and, if willing to receive further information, are then referred to HvS and/or TB to receive detailed study information and to evaluate eligibility. They then have a minimum of one week before they are contacted to inquire if they have any remaining questions and if they are willing to participate.

We include patients with a radiculopathy due to, MRI confirmed, single level soft disc herniation at the C5-C6 or C6-C7 level, refractory to at least 12 weeks of conservative treatment. They must be able to actively make a full flexion to extension movement. If there is osseous degeneration at the target level a flexion/extension radiograph is made to be certain that the segment is not spontaneously fused, which is defined as cortical bridging or less than 2° segmental range of motion. At the time of conception of the study there was insufficient information about safety in patients with myelopathy; patients with a myelopathy are therefore excluded. Other reasons for exclusion are degenerative changes at more than the target level, prior surgery or radiotherapy to the cervical spine, active infection, or a history of malignancy. Inclusion and exclusion criteria are depicted in Additional file 1: Table S1.

Informed consent will be acquired from all participants. The study has been approved by the local institutional medical ethical committee (Medical Research Ethics Committee Maastricht UMC+, METC 06-1-098) and has been registered on clinicaltrials.gov (NCT00868335).

### Sample size calculation

Sample size calculation is based on the presence or absence of fusion at the operated segment, as the primary outcome is expected to be directly correlated to the presence of fusion. Fusion is defined as < 2° range of motion. At an α set at 0.05, the power (β) of 80% to detect a 40% difference in the amount of patients with < 2° range of motion, the necessary number of participants per group is ten. At a projected loss to follow up of 20% the amount of participants needed is twelve in each group. We also include a pilot group of three patients that will all receive CDA, which will precede the randomised groups, to limit the possible influence of a learning curve. The total number of patients that will be included is therefore 27.

### Patient allocation and blinding

Each patient is allocated to either arthroplasty or simple discectomy by randomly drawing an unmarked opaque sealed envelope from a non-sequential stack. Randomization is performed in a 1:1 ratio without a fixed block size. The patient is informed about the result of the randomisation at the first radiological examination moment, up to two days before surgery. The surgeon and other caregivers are not blinded. Assessment of the radiological data is not blinded, since absence or presence of a prosthesis is clearly visible. The clinical outcome data are collected by a blinded caregiver.

### Interventions

All patients are operated in the same centre by a single experienced spine surgeon. The prosthesis used is the Activ-C (B. Braun Aesculap, Germany).Surgery is performed under general anaesthesia, with the patient in a supine position and the neck in a neutral position to slight extension. Antibiotic prophylaxis is given at induction of anaesthesia. The location of the incision is verified using fluoroscopy. In case of poor visibility of the target level, the shoulders are retracted by elastic tape. A transverse incision is made on the right side of the neck. The anterior aspect of the cervical spine is approached medially to the carotid sheath. The level is verified during surgery using fluoroscopy. The vertebral bodies are then distracted using two distraction pins and a Caspar spreader. Using a microscope, a standard discectomy is performed. The posterior longitudinal ligament is opened to reveal any sequesters. Decompression is verified by probing of the neuroforamen. In the simple discectomy group haemostasis is checked, a low-vacuum drain is placed and the wound is closed in two layers.

In the arthroplasty group the correct prosthesis size is chosen using a fitting-prosthesis, the position is controlled by lateral en anterior–posterior fluoroscopy, taking care to place it in the midline. Subsequently, the caudal endplate is prepared for the keel of the prosthesis and the prosthesis is placed. The definite position is checked using fluoroscopy in 2 directions. The wound is rinsed to clear any drilling material, haemostasis is checked, a low-vacuum drain is placed and the wound is closed in two layers.

### Post-surgical care

Antibiotic prophylaxis is continued for 24 hours in the arthroplasty group. Cervical spine X-rays are acquired the day after surgery and the wound drain is removed. Patients in both groups are encouraged to mobilise, and resume home activities and work as soon as possible. A collar is not prescribed and patients are not routinely referred to a physical therapist. Patients in the arthroplasty group are prescribed indomethacin 50 mg three times a day for a period of three weeks to prevent periprosthetic calcifications, unless they have a history of gastric or intestinal complaints, or previous adverse reactions to non-steroidal anti-inflammatory drugs (NSAIDs). Patients in the simple discectomy group are allowed to use NSAIDs.

### Radiological outcome measures

Radiological outcome measures are evaluated pre-surgery (0–2 days), and at three and twelve months post-surgery. Radiological outcome measures are:

- Absence or presence of normal sequence of segmental contributions to cervical spine movement, analysed through fluoroscopic flexion-extension recording (FFER). The method of data acquisition and analysis is described below.
- Segmental range of motion (sROM) of the target level is measured.
- The presence of fusion is defined by cortical bridging and/or sROM less than < 2°.

### Radiological data acquisition

Fluoroscopic recordings are made with a digital X-ray detector (Toshiba Infinix VC-i, Toshiba Medical Systems Corporation, Japan), capturing frames of 1024 × 1024 pixels, at 10 frames per second. The recordings are stored without compression. To acquire the FFER, the subject is placed on a stool, with his shoulders perpendicular to the digital x-ray receptor plate. The shoulder nearest to it is placed directly against it. The subject is asked to move the head in maximal extension without moving the upper part of his body. As soon as the recording is started, the subject is instructed to move the head in the sagittal plane from maximal extension to maximal flexion, without moving the upper part of the body. The subject is then allowed to relax for a moment, and then the movement from maximal flexion to maximal extension is recorded. The subject is asked to perform each movement in about five to ten seconds. It is important that the subject’s shoulders are kept as low as possible during fluoroscopy to ensure that all the cervical vertebrae are visible.

### Radiological data processing

The authors have developed custom software that uses image recognition algorithms to track the skull and cervical vertebrae during flexion and extension throughout these series of frames [16]. The software follows bony structures within user-defined template areas (splines) throughout all frames, using a best-fit principle to match normalized gradient field images. To define these template areas the user of the software draws polygons around all vertebrae and part of the skull on the median frame of the recording (Additional file 2: Figure S1). After the software has completed tracking these structures they can be manually evaluated, and corrections can be made if necessary.

The rotational data between frames for each bony structure enables the user to calculate sROMs, but also sagittal rotation within a motion segment through time. The sequence of various segmental contributions to movement of the entire cervical spine can therefore be established (Additional file 3: Figure S2). The sequence of segmental contributions is evaluated during full ‘bending’ extension-to-flexion, and vice versa. It is compared to ten healthy controls, and a historical control group [13-15].

### Clinical outcome measures

At the same three time points at which the radiological analysis takes place the subject will be neurologically examined by an independent physician or physician assistant who is blinded to the treatment group. Muscle strength in myotomes C5-Th1 according to the Medical Research Council (MRC) Scale for Muscle Strength, sensory deficits in dermatomes C5-Th1, and deep tendon reflexes, are determined. Cervical spine motion is also estimated (flexion, extension, rotation, and lateral bending, estimated in degrees relative to the neutral position, i.e. the ears are aligned directly over the shoulders). Adverse events (such as infection and re-operation) are registered, and the patients are asked to fill in the following questionnaires:

- Visual analogue scale for pain radiating to the arm (VAS-arm), on a 100 mm scale, ranging from 0 (no pain at all) to 100 (worst pain imaginable).
- Visual analogue scale for neck pain (VAS-neck), on a 100 mm scale, ranging from 0 (no pain at all) to 100 (worst pain imaginable).
- Neck disability index (NDI), a 10-item questionnaire with a maximum score of 50 points (0 points being no perceived disability, and 50 points the maximum amount of disability), to assess perceived disability in work related, and non-work related activities due to neck complaints.
- Short-form 36 (SF36), a survey to assess general health, by using 36 questions to score 8 sections; physical functioning, social functioning, bodily pain, general health, vitality, mental health, and problems with daily activities due to either physical or emotional complaints.

At the two postoperative time points (three and twelve months) one additional outcome is measured:

- Odom’s outcome scale, a four-level outcome scale rating patients’ outcome from poor to excellent depending on the level of resolution, improvement, or persistence of pre-operative symptoms.

### Statistical methods

Dichotomous outcome measures (presence of normal sequence of segmental contributions, presence of fusion), and ordinal outcome measures (Odom’s outcome scale) will be analysed using a χ2 analysis. Statistical significance will be defined as P < 0.05 in the χ2 analysis. When the requirements of the χ2 analysis are not met (i.e. less than 5 samples per field) Fisher's exact test will be used. Continuous outcome measures (NDI, VAS, SF36, sROM) will be analysed using an independent samples t-Test, after testing for normality. Statistical significance will be defined as P < 0.05 in the t-Test. If normality is not met, a Mann–Whitney *U* test will be used. All data will be analysed according to the ‘intent-to-treat’ principle, additionally a sensitivity analysis will be performed, based on presence or absence of fusion.

## Discussion

The role of segmental fusion as a cause of ASDis, is widely discussed, and as the intended purpose of arthroplasty is prevention of ASDis, so is the indication for this type of treatment [6,9,17]. Given the fairly low incidence of ASDis, which has been reported to be about 2.9% annually, large studies with long term follow up are necessary to be able to determine the efficacy of arthroplasty to in fact prevent ASDis [8,18]. As an alternative to these studies the authors decided to investigate the premise itself, that arthroplasty does in fact restore/preserve a normal sequence of segmental contributions in the cervical spine, while simple discectomy is likely to do not. Although a fused segment (in any of the two groups) will have no significant contribution, the sequence of the remaining segments is still of use. As this gives information about the influence of a fused segment on the rest of the cervical spine, and if there is a larger disturbance than simply a missing peak.

Many studies investigating arthroplasty determine mobility of the target level by measuring sROM before and after surgery, which allows for the conclusion that there is mobility, but gives no information other than the quantity of mobility. The authors therefore chose to use a method that was previously used in healthy controls to describe the dynamic process of cervical spine mobility in more detail, using the sequence of segmental contributions. This sequence has been reported to be much more consistent than sROMs, which are limited by high intra- and inter-individual variability [13-15]. Since the results are more consistent, sample size can be lower. No results from a randomized controlled trial about the prosthesis used have been reported to date. Clinical outcome measures were therefore also included as secondary outcome measures, to evaluate if clinical results are in line with previous reports about other cervical disc prostheses.

As in almost all surgical studies, caregivers and X-ray analyses cannot be blinded, and patients report their own outcomes, therewith invoking risk of bias. This is a common weakness in this type of study, as blinding patients in these types of device studies is often regarded as not ethically appropriate, and most of the clinical outcomes are patient-reported outcomes [10]. As a result, many of the outcomes may suffer from bias, although the differences that have been reported so far were usually small [10]. The radiological outcome cannot be blinded completely since the prosthesis is clearly visible in the FFER, and although the motion analysis is performed by image recognition software, these analyses are checked manually to avoid errors. The clinical outcome data are collected by a blinded caregiver; since the patient is not blinded the possibility of the caregiver receiving information about the surgery is therefore present. Sample size is small in this study. However, given the large expected difference in the amount of fusion between the groups, and the consistent findings when analysing sequence of segmental contributions, the authors expect to be able to draw conclusions about the primary outcome. For the secondary outcomes the sample size will be too small. However, the authors’ haven previously shown that clinically relevant differences are not to be expected, and only aim to see if results are in line with those described in literature [10].

The study is industry sponsored, as are most of the RCTs on this subject [10]. This also can lead to bias. The influence of a conflict of interest on outcomes in trials investigating CDA has recently been investigated, which led to the conclusion that while there was no influence on quality of life outcomes, it was associated with lower reported complication rates [12]. In the current study the sponsor has no influence on planning or conducting the study, no influence on data acquisition or analysis, or any aspect of the publication process.

The study has several strengths. First, the inclusion criteria are very strict. Inclusion of subjects with multiple degenerated segments would make it hard to distinguish between effects on the target level and adjacent levels due to the surgery, or due to pre-existent degenerative changes. Inclusion of only single level cases, without degenerative changes to the other levels, will therefore minimize confounding. Second, the method of radiological analysis gives more information than the traditionally made function radiographs. This allows for a more thorough analysis of mobility of the target level, as well as motion patterns of the cervical spine. This enables a clearer conclusion if cervical spine motion can be defined as physiological after arthroplasty, when compared to simple discectomy. As restoring normal mobility is the premise of the possible reduction of ASD through use of CDA, this method of analysis therefore allows for and earlier indication if a specific type of prosthesis might be able to reduce ASD in the future.

## Additional files

Additional file 1: Table S1. Inclusion and exclusion criteria for the RCT. Additional file 2: Figure S1. Template area example; example of user defined template areas on the median frame of a FFER of a subject with cervical degenerative disc disease. Additional file 3: Figure S2. Example graph; example graph depicting sagittal rotation in motion segments in the lower cervical spine during movement from maximal extension to maximal flexion in C_4_-C_7_ in an asymptomatic subject. Peaks in the graph depict maximum contributions of that motion segment at that moment in the flexion movement. From right to left, a peak in C_6_-C_7_ is followed by a peak in C_5_-C_6_, and then C_4_-C_5_. This is then followed by a second peak in C_5_-C_6_, and then in C_6_-C_7_. According to historical data this sequence is commonly seen in healthy controls.

## Abbreviations

RCT: Randomized controlled trial
CDA: Cervical disc arthroplasty
ASD: Adjacent segment degeneration
ASDeg: Adjacent segment degeneration
ASDis: Adjacent segment disease (symptomatic adjacent segment degeneration)
FFER: Fluoroscopic flexion-extension recording
sROM: Segmental range of motion
MRI: Magnetic resonance imaging
MRC: Medical research council
NDI: Neck disability index
SF36: Short form 36
VAS: Visual analogue scale
NSAID: Non-steroidal anti-inflammatory drug
