# Supplementary figures and images for: Cervical spine kinematics after anterior cervical discectomy with or without implantation of a mobile cervical disc prosthesis; an RCT
Source: BMC Musculoskelet Disord. 2015 Feb 21;16:34. doi: 10.1186/s12891-015-0479-4 (PMC4349598; doi:10.1186/s12891-015-0479-4)

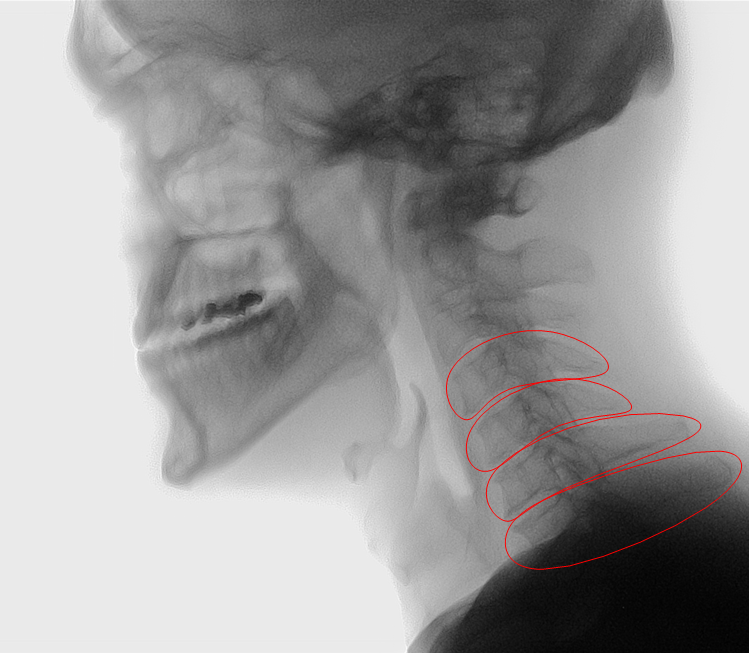

Supplement: Additional file 2: Figure S1. — Template area example; example of user defined template areas on the median frame of a FFER of a subject with cervical degenerative disc disease. [file 12891_2015_479_MOESM2_ESM.png]

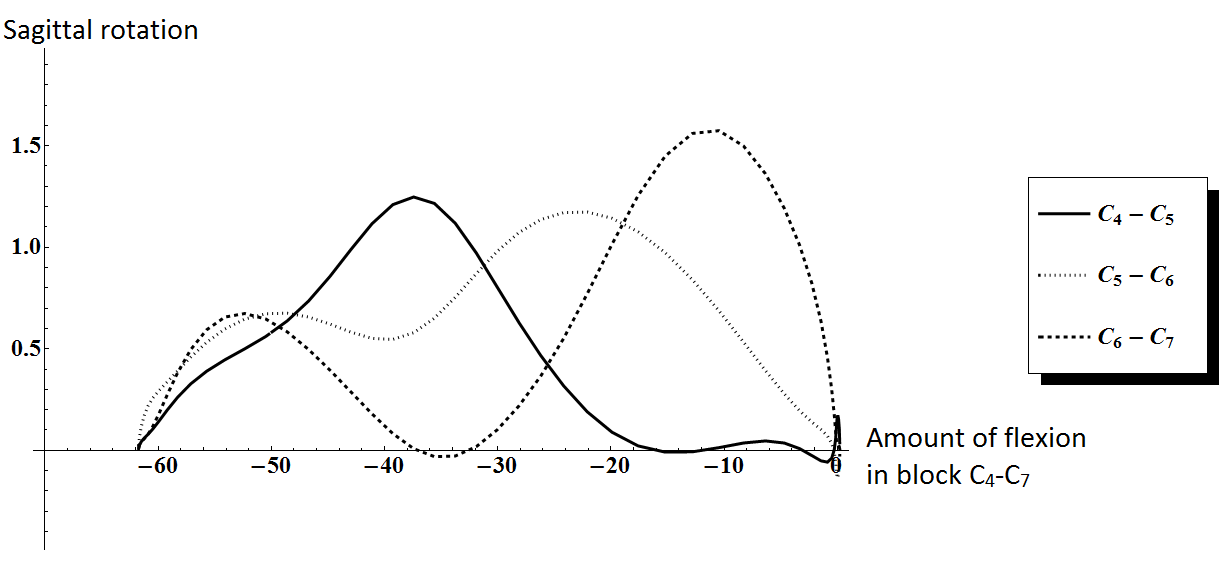

Supplement: Additional file 3: Figure S2. — Example graph; example graph depicting sagittal rotation in motion segments in the lower cervical spine during movement from maximal extension to maximal flexion in C4-C7 in an asymptomatic subject. Peaks in the graph depict maximum contributions of that motion segment at that moment in the flexion movement. From right to left, a peak in C6-C7 is followed by a peak in C5-C6, and then C4-C5. This is then followed by a second peak in C5-C6, and then in C6-C7. According to historical data this sequence is commonly seen in healthy controls. [file 12891_2015_479_MOESM3_ESM.zip › 12891_2015_479_MOESM3_ESM.bmp]
